# Persistence of Inflammatory Response to Intense Exercise in Diabetic Rats

**DOI:** 10.1155/2012/213986

**Published:** 2012-08-13

**Authors:** José Ricardo Bortolon, Antonio José de Almeida Silva Junior, Gilson Masahiro Murata, Philip Newsholme, Rui Curi, Tania Cristina Pithon-Curi, Elaine Hatanaka

**Affiliations:** ^1^Institute of Physical Activity and Sport Sciences, Cruzeiro do Sul University, Rua Galvão Bueno, 868, 13° andar, 01506-000 São Paulo, SP, Brazil; ^2^Institute of Biomedical Sciences, University of São Paulo, 05508-900 São Paulo, Brazil; ^3^School of Biomolecular and Biomedical Science, Conway Institute, University College Dublin, Dublin, Ireland; ^4^School of Biomedical Sciences, Curtin University, Perth, WA 6000, Australia

## Abstract

In this study we evaluated the onset and resolution of inflammation in control and streptozotocin-induced diabetic rats subjected to a single session of intense exercise. The following measurements were carried out prior to, immediately after, and 2 and 24 hours after exercise: plasma levels of proinflammatory cytokines (TNF-*α*, IL-1*β*, IL-6, CINC-2*α*/*β*, MIP-3*α*, and IL-6), immunoglobulins (IgA and IgM), acute phase proteins (CRP and C3), and creatine kinase (CK) activity. We also examined the occurrence of macrophage death by measurements of macrophages necrosis (loss of membrane integrity) and DNA fragmentation. An increase was observed in the concentration of IL-1*β* (3.3-fold) and TNF-*α* (2.0-fold) and in the proportion of necrotic macrophages (4.5-fold) in diabetic rats 24 hours after exercise, while the control group showed basal measurements. Twenty-four hours after the exercise, serum CK activity was elevated in diabetic rats but not in control animals. We concluded that lesion and inflammations resulting from intense exercise were greater and lasted longer in diabetic animals than in nondiabetic control rats.

## 1. Introduction

Moderate and regular aerobic exercise reduces insulin resistance and improves antioxidant and immune capacities, preventing the onset and progression of chronic diseases that present low-grade systemic inflammation (e.g., diabetes mellitus) [1–5]. On the other hand, high-intensity exercise imposes major acute endocrine, metabolic, and immune changes that may persist for a period of several hours after the exercise and may be detrimental to the health of diabetic patients [6]. The American College of Sports Medicine (ACSM), the American Diabetes Association (ADA), the European Association for the Study of Diabetes (EASD), and the American Heart Association (AHA) recommend regular aerobic and resistance exercising for patients with diabetes without major complications [7–10]. However, their guidelines do not specify the intensity or the most suitable type of physical exercise to maximize the benefits of exercise with minimal risk while ensuring the proper regulation of immune function and inflammatory control. Furthermore, there are no studies that have determined the time required for diabetic patients to recover from exercise-induced skeletal muscle injury.

Strenuous physical exercise can result in muscle injury, promoting the release of skeletal muscle enzymes creatine kinase (CK) and lactate dehydrogenase (LDH) [11]. Inflammatory response begins by infiltration of fluid, plasma proteins, and leukocytes (e.g., macrophages) into the injured muscle. Certain cytokines (e.g., TNF-*α*, IL-1*β*, IL-6, and CINC-2*αβ*) play a key role in the onset of inflammatory response [12]. Exercise-induced muscle injury is also associated with elevation in the production of acute phase proteins such as C-reactive protein (CRP) and complement component 3 (C3). CRP, C3, and immunoglobulins such as immunoglobulin A (IgA) and immunoglobulin M (IgM) act as opsonins and are important in identifying and neutralizing bacteria and viruses during acute phase response [13]. Acute phase response ends when the blood levels of inflammatory markers decrease. Chronic inflammation is characterized by increased levels of acute phase markers and proinflammatory proteins in serum for a long period of time.

Inflammation has been associated with the progression of diabetic vascular complications such as retinopathy [14], nephropathy [15], neuropathy [16], and atherosclerosis. Insulin resistance is associated with a state of chronic low-grade inflammation, and mediators such as tumor necrosis factor-*α* (TNF-*α*), interleukin 1beta (IL-1*β*), and interleukin-6 (IL-6) have been identified as being involved in metabolic control and impairment in insulin-receptor signaling [17]. Hence, the determination of optimal markers of lesion and inflammation that appear in blood during physical effort may be important in determining the appropriate intensity of exercise for people with a chronic pro-inflammatory status, such as diabetes. Additionally, it is important to determine the optimal time required to recover from exercise-induced tissue damage.

This study was designed to determine the effects of an intense exercise session on blood levels of glucose, IL-1*β*, TNF-*α*, IL-6, cytokine-induced neutrophil chemoattractant 2 (CINC-2*αβ*), C-reactive protein (CRP), macrophage Inflammatory protein (MIP-3*α*/*β*), immunoglobulin A (IgA), immunoglobulin M (IgM) and complement component 3 (C3) in streptozotocin-induced diabetic rats and nondiabetic control rats. The proportion of necrotic (loss of plasma membrane integrity) and apoptotic (DNA fragmentation) macrophages was also evaluated. These parameters were determined in diabetic rats and nondiabetic controls before and immediately after an intense exercise session on a treadmill, and 2 and 24 hours thereafter.

## 2. Materials and Methods

### 2.1. Animals

Male Wistar rats (180 ± 20 g) were kept in a room with an inverted 12-hour light/dark cycle under standardized conditions of temperature and humidity. Standard lab animal feed (52% carbohydrates, 21% proteins, and 4% lipids; Nuvilab CR1-Nuvital) and water were provided *ad libitum*. The experiment was approved by the Ethical Committee of the Cruzeiro do Sul University (Protocol no. 017/2009) and was performed according to the Guidelines for the Care and Use of Laboratory Animals. The animals were divided into eight groups with at least seven animals per group: (i) control nonexercised (0), (ii) control immediately after exercise (IA), (iii) control 2 h after the exercise (2 h), (iv) control 24 h after the exercise, (24 h) (v) diabetic nonexercised (0), (vi) diabetic immediately after exercise (IA), (vii) diabetic 2 h after the exercise (2 h), and (viii) diabetic 24 h after the exercise (24 h). 

 Fast experimental design: experimental type 1 diabetes was induced, and the diabetic state was confirmed 48 h after the streptozotocin injection. One week after the induction of diabetes, the rats were adapted to the treadmill for another week. After the period of adaptation, the nonexercised groups (diabetes and control) remained in the cages (four animals *per cage*), and the exercised animals were subjected to a single session of intense exercise. Blood samples were drawn prior to, immediately after, and 2 and 24 hours after exercise. Blood samples were drawn from decapitated rats into BD Vacutainer tube containing EDTA, which was used for plasma collection.

### 2.2. Induction of Diabetes

Experimental type 1 diabetes was induced by intraperitoneal injection of 65 mg/kg b.w. of streptozotocin dissolved in citrate buffer (pH 4.2). The diabetic state was confirmed 48 h after the streptozotocin injection by blood glucose levels exceeding 250 mg/dL, which were estimated using a glucose meter (Roche, São Paulo, Brazil) [18]. Blood samples were drawn from a cut at the tip of the animal's tail. The physical adaptation training was initiated 7 days after the induction of diabetes.

### 2.3. Physical Adaptation Training Protocol

The physical adaptation training was initiated seven days after the induction of diabetes [5]. The rats were adapted to the treadmill for 7 days. This adaptation consisted of daily exercise training for 15 min at a speed of 0.3 km/h. One day after the period of adaptation, the nonexercised group remained in the cages, and the exercised animals were subjected to a single session of intense exercise.

The initial speed of the treadmill was 0.3 km/h. At 3-minute intervals, the speed was increased by 0.3 km/h until 20 min of exercise training were reached [19]. A resulting VO_2max⁡_  intensity of up to approximately 70% was reached, which is consistent with our earlier studies [19].

Before (the nonexercised group) and after completion of the exercise, and 2 and 24 hours after the intense training program, the animals were killed, and blood and macrophage samples were collected. Plasma was collected and stored at −80°C before determining cytokines, growth factors, acute phase proteins, immunoglobulins, and glucose levels.

### 2.4. Determination of Plasma Interleukin Levels

Plasma levels of IL-6, IL-1*β*, TNF-*α*, CINC, and MIP-3 were determined by enzyme-linked immunosorbent assay (ELISA), using a DuoSet Kit (Quantikine, R&D Systems, Minneapolis, MN, USA) according to the manufacturer's instructions. 

### 2.5. Determination of Serum C-reactive Protein (CRP), Complement Fraction 3 (C3), and Immunoglobulin Levels

CRP, C3, IgA, and IgM were determined by a highly sensitive immunoturbidimetric method (Bioclin Diagnostics, São Paulo, Brazil), following the manufacturer's instructions. 

### 2.6. Determination of Creatine Kinase (CK) Activity

A Bioclin Diagnostics kit (São Paulo, Brazil) was used, and the measurements were taken following the manufacturer's instructions. The equipment was calibrated for each determination, and a control serum was used to validate the data obtained.

### 2.7. Preparation of Peritoneal Macrophages

Macrophages were obtained by intraperitoneal (i.p.) lavage with 40 mL sterile phosphate-buffered saline (PBS), immediately after rats' decapitation and blood collection. The peritoneal fluid was centrifuged (400 g for 10 min) and washed twice with PBS and cells were counted in a Neubauer chamber using an optical microscope (Alphaphot-2, Nikon, Japan) and trypan blue solution (at 1% in PBS). Macrophage loss of membrane integrity (necrosis) and DNA fragmentation (apoptosis) was immediately determined. 

### 2.8. Cell Viability Assay (Proportion of Necrotic Cells)

Peritoneal macrophage viability was assessed using a FACSCalibur Cytometer (Becton Dickinson Systems, San Jose, CA, USA). Briefly, after the collection, the cells (1.0 × 10^6^ cell/mL) were threaded with propidium iodide stain (solution at 0.05% in PBS). The percentage of viable cells in each sample was immediately analyzed. Propidium iodide (PI) fluorescence was measured using the FL2 channel (orange-red fluorescence) [20]. Ten thousand events were analyzed per sample. 

### 2.9. Proportion of Cells with Fragmented DNA (Apoptotic Cells)

DNA fragmentation was analyzed by flow cytometry after DNA staining with PI. The presence of detergent in the solution permeabilized the cells, which promptly incorporated the dye into DNA. Briefly, after the collection, the cells were centrifuged at 400 ×g for 15 min at 4°C. The resulting pellet (1.0 × 10^6^ cell) was carefully resuspended in 300 *μ*L hypotonic solution containing 50 *μ*g/mL propidium iodide, 0.1% sodium citrate, and 0.1% Triton X-100. The cells were then incubated for 2 h at 4°C. Fluorescence was measured and analyzed as described above [20].

### 2.10. Statistical Analysis

The values are presented as mean ± standard error of at least seven animals per group. The statistical analysis consisted of one-way Analysis of Variance (ANOVA) using the Student-Newman-Keuls post hoc Multiple Comparison test (INStat; Graph Pad Software, San Diego, CA, USA). The level of significance was set at *P* < 0.05.

## 3. Results

### 3.1. Blood Glucose

**Figure 1:**
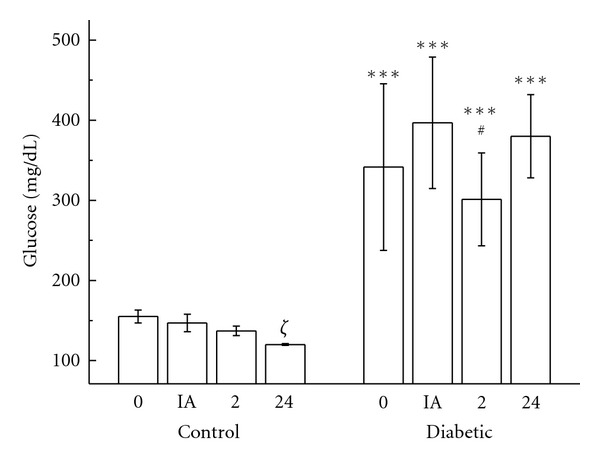
Blood concentrations of glucose (mg/dL) in controls and diabetic rats measured before (0), immediately after (IA), 2 h (2), and 24 h (24) after exercise. The values are presented as mean ± SD of 7 animals per group. ***P < 0.001 for comparison of the diabetic and control groups in same condition of time. *ξ*_*P*_ < 0.05 for comparison of the control groups prior to exercise and 24 h after exercise, and ^#^ *P* < 0.05 for comparison of the diabetic groups prior to exercise and 2 h after exercise.

Blood glucose was measured before, immediately after, 2 h, and 24 h after the intense treadmill exercise to ascertain if the exercise protocol was able to modify glycemia in nontrained diabetic and control rats. Blood glucose levels in control rats decreased immediately, 2 h, and 24 h after the exhaustive treadmill exercise. In the control group, intense exercise was found to diminish serum glucose levels over the 24-hour postexercise period. However, the glucose levels of diabetic rats remained higher at all the evaluated times than in the control group. Nevertheless, the blood glucose levels of the diabetic animals 2 hours after exercise showed a 24% decrease when compared with their levels immediately after the exercise (Figure 1).

### 3.2. Plasma Interleukin, Immunoglobulin, and Complement C3 Levels

**Figure 2:**
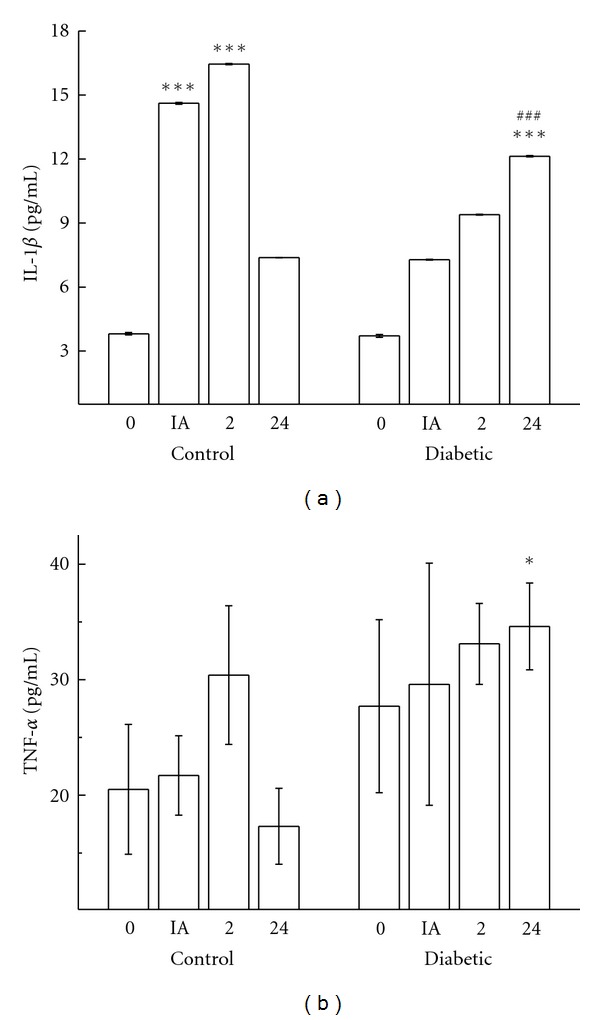
Blood concentrations of IL-1*β* (a) and TNF-*α* (pg/mL) (b) in controls and diabetic rats measured before (0), immediately after (IA), 2 h (2), and 24 h (24) after exercise. The values are presented as mean ± SD of 7 animals per group. *P < 0.05 for comparison of control and diabetic groups 24 h after exercise, ***P < 0.001 for comparison of the control and diabetic groups in same condition of time, and ^###^ *P* < 0.001 for comparison of the diabetic groups prior to exercise and 24 h after exercise.

Plasma IL-1*β* increased immediately (*P* < 0.001) and 2 hours (*P* < 0.001) after exercise in the control animals, but declined after 24 hours. In diabetic rats, plasma IL-1*β* levels increased (3.3-fold) for up to 24 hours after the exercise (Figure 2(a)). The same response pattern was observed for TNF-*α*. Twenty-four hours after exercise, diabetic rats still showed augmented concentrations of TNF-*α* (2.0-fold), while the control group returned to basal values (Figure 2(b)). 

**Figure 3:**
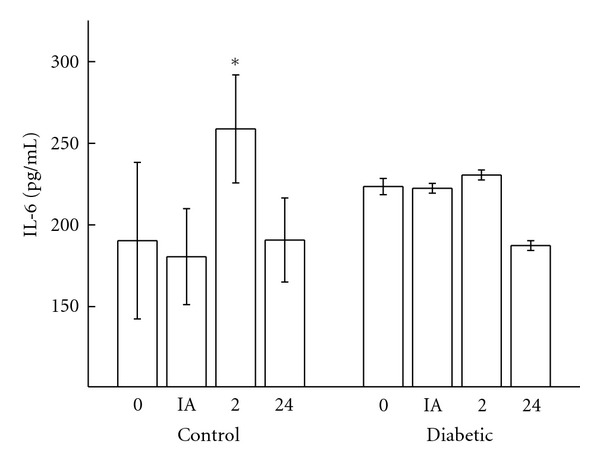
Blood concentrations of IL-6 (pg/mL) in controls and diabetic rats measured before (0), immediately after (IA), 2 h (2), and 24 h (24) after exercise. The values are presented as mean ± SD of 7 animals per group. *P < 0.05 for comparison of the control group before exercise and 2 hours after exercise.

Plasma IL-6 levels peaked two hours after the exercise session in control rats. The diabetic group showed a relatively stable plasma concentration of IL-6 with no significant difference at any point in time after the exercise session (Figure 3). 

Under the conditions of this study, the diabetic and control groups showed no significant difference in serum levels of CINC-2*α*/*β*, MIP-3*α*, CRP, C3, IgA, IgM, and C3 at any point in time after the exercise session (Table 1).

### 3.3. Proportion of Necrotic Cells and Cells with Fragmented DNA

**Figure 4:**
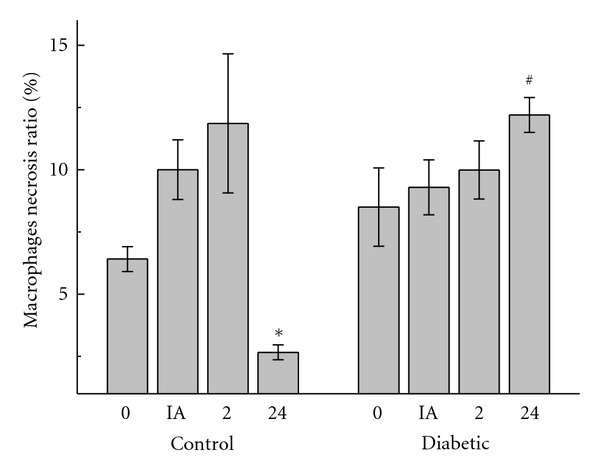
Number of**  **macrophages with signs of necrosis (loss of plasma membrane integrity) in the peritoneal cavity in controls and diabetic rats measured before (0), immediately (IA), 2 h (2), and 24 h (24) after exercise. The values are presented as mean ± SD of 7 animals per group. *P < 0.05 for comparison of the control group before exercise and 24 h after exercise, and ^#^ *P* < 0.05 for comparison of diabetic and control groups 24 h after exercise.

Necrotic macrophage death was significantly augmented in control rats immediately after and 2 hours after the intense exercise session, but returned to basal levels after 24 hours. However, necrotic macrophage death in diabetic rats increased after the exercise session up to the 24-hour time point (Figure 4). Macrophage apoptosis did not differ significantly between groups or time points following the exercise session (data not shown). 

### 3.4. Creatine Kinase Activity

**Figure 5:**
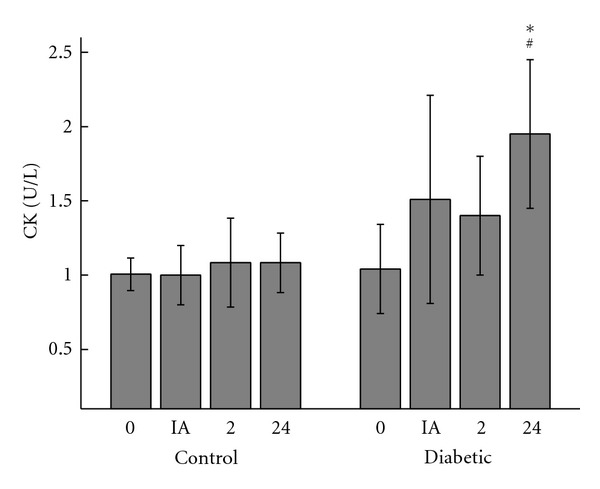
Serum activity of CK (U/L) in controls and diabetic rats measured before (0), immediately after (IA), 2 h (2), and 24 h (24) after exercise. The values are presented as mean ± SD of 7 animals per group. *P < 0.05 for comparison of the diabetic and control groups in same condition of time and ^#^ *P* < 0.05 for comparison of the diabetic groups prior to exercise and 24 h after exercise.

Twenty-four hours after exercise, diabetic rats showed increased serum CK activity, while the control group showed no change from basal conditions (Figure 5).

## 4. Discussion

This paper is the first to report that, after an intense exercise session, inflammatory markers in diabetic rats were augmented in comparison to nondiabetic animals. Intense exercise reduced serum glucose levels for at least 24 hours after exercise in the control group. Muscle contraction, *per se*, interacts with the insulin signaling pathway to GLUT4 translocation, allowing for enhanced insulin sensitivity and thus glucose uptake [21, 22]. Previous studies involving resistance and aerobic exercise training have described improved glycemic control in diabetic and nondiabetic groups, providing a clear rationale for recommending exercise for diabetic patients. Other beneficial effects of exercise include improvement of immune function and leukocyte activation [6], alteration of lipid profile and cardiovascular risks [23], increased motor control [24], diminished risks of neurodegenerative diseases [25], and improvement of vascular flux and vascular response [26].

However, exercise accompanied by lesion/inflammation may be detrimental to the health of diabetic patients. The guidelines currently used for prescribing exercise for diabetics do not provide information on the intensity for specific subpopulations based on their inflammatory profile. Moreover, little is known about the time required for diabetics to recover without risks from exercise-induced tissue damage. Few studies have focused on the intensity to be considered for exercise programs for diabetic patients. Kawaji et al. [27], Fujita et al. [28], and Belli et al. [29] demonstrated that determination of the anaerobic threshold is useful in establishing the intensity of exercise suitable for diabetes mellitus type 2 patients.

Metabolically exhausted muscle fibers exhibit a decrease in membrane resistance and associated CK and LDH release into plasma [11]. In the conditions of this study, 24 hours after exercise, diabetic rats—but not control animals—showed increased serum CK activity. Exercise-induced lesions cause the levels of pro-inflammatory cytokines such as TNF-*α*, IL-1*β*, and IL-6 to increase by two- to threefold [30]. This increase is accompanied by an augmentation in serum levels of acute phase proteins such as C-reactive protein and C3. CRP is the main human acute-phase protein. The biological function of CRP appears to be defense of the host against bacterial pathogens. CRP interacts with Fc receptors on phagocytic cells and, like C3, acts as an opsonin [31]. Other immune markers that undergo changes according to the type/duration of exercise are immunoglobulins. Our results indicated that 24 hours after intense exercise, the diabetic rats exhibited increased concentrations of IL-1*β* and TNF-*α* and a higher proportion of necrotic diabetic macrophages, while these mediators returned to resting levels in the controls. The exact reason for these results remains unclear, but the idea that wound healing and leukocyte functions are altered in diabetes is supported by data [14, 15, 32, 33]. Strenuous exercise causes muscle damage. Muscle tissue repair is a process that involves cellular, immunological and biochemical events that lead to tissue restoration and involve several components such as platelets, resident cells (fibroblasts, keratinocytes, endothelial, and nervous cells), leukocytes (macrophages, neutrophils and lymphocytes), lipid mediators such as prostanoids (prostaglandin, thromboxanes, and prostacyclins), protein mediators (acute phase proteins, cytokines, and growth factors), reactive oxygen, and nitrogen species. The difficulty of muscle repair in diabetics may be due to factors such as the presence of glycated proteins (AGEs), which not only activate immune cells but also alter the functions of other cell types; decreased perfusion rates, especially in the lower limbs, peripheral neuropathy, which reduces sensitivity to pain, facilitating the emergence and progression of an injury; increased oxidative stress which may induce inflammation and cell death; impairment of leukocyte functions [14]. Additionally, the neutrophils of diabetic patients show alterations in almost all cell functions, such as decreased migration to inflammatory sites, phagocytosis, release of lytic proteases, production of reactive oxygen species, apoptosis, and increased cytokine production [33]. These alterations may influence the inflammatory phase of muscle repair in diabetics, contributing to the effects observed. In diabetes, the inflammatory phase of tissue repair begins slowly, but persistent inflammation is a common finding in chronic wounds of diabetics [32, 33].

Increased levels of inflammatory mediators such as pro-inflammatory cytokines have been reported in diabetes to be a consequence and/or cause of hyperglycemia. Pro-inflammatory cytokines and free fatty acids (FFA) have been considered the link between inflammation and insulin resistance [33]. At the molecular level, the exposure of cells to TNF-*α*, IL-1*β*, or IL-6 stimulates inhibitory phosphorylation of serine residues of insulin receptor. This effect is directly involved in insulin resistance. Additionally, abnormal levels of pro-inflammatory cytokines participate in the development of vascular complications and increasing susceptibility to invasive microorganisms. Our group recently demonstrated that moderate exercise improves leukocyte function and decreases inflammation in diabetic rats [5]. We demonstrated that a moderate three-week exercise regimen on a treadmill decreases serum levels of TNF-*α*, CINC-2*α*/*β*, IL-1*β*, IL-6, CRP, and FFA in diabetic rats when compared to sedentary diabetic animals. Exercise also attenuated the increased responsiveness of leukocytes in diabetic patients when compared to those of controls, diminishing ROS release by neutrophils and macrophages [5].

Exercise-induced lesions cause the levels of proinflammatory cytokines such as TNF-*α*, IL-1*β*, and IL-6 to increase two- to threefold. However, exercise itself may also result in augmented release of IL-6 into the circulatory system without causing inflammation or lesion. Muscle fibers themselves may be a source of IL-6 production during exercise. In our study, we observed an increase in the TNF-*α* and IL-1*β* levels, but not in IL-6 [13].

The combined effect of an increment in the production of TNF-*α* and IL-1*β* in diabetic patients following an intense exercise session may elevate the inflammatory response, contributing to induce a hyperglycemic state and to the onset of diabetic complications [32, 34]. These mediators alter homeostasis, cause hyperglycemia, activate macrophages, contribute to tissue damage, and increase susceptibility to invasive microorganisms. Several studies indicate that inflammation plays a role in the progression of diabetic microvascular complications such as retinopathy, nephropathy, and neuropathy and macrovascular complications, that is, atherosclerosis. TNF-*α*, IL-1*β*, and CINC-2*α*/*β* have important effects on immune cells. At lower levels, these cytokines are important for the activation of endothelial cells and the expression of ligands for adhesion molecules for leukocyte integrins and endothelial selectins. Chronic inflammation results in fibrosis and loss of organ function, negatively affecting the health of diabetics. Furthermore, intense exercise in diabetics can also increase serum levels of hyperglycemic hormones such as adrenalin, cortisone, and glucagon [22]. 

Evidence supports the idea that intense exercise induces immune suppression and increases susceptibility to infection. Recently we took extensive measurements of systemic inflammation and neutrophil function in athletes after a competitive match. We concluded that playing induces inflammation, activates neutrophils, and reduces the efficiency of neutrophils against infection resulting from exposure to pathogens immediately after a match [35]. For this reason, studying muscle lesions and inflammation after exercise may be an important strategy to establish a suitable intensity of exercise for people with a chronic pro-inflammatory status, such as elderly, obese, diabetic, cancer, arthritis, and Alzheimer's patients, and people suffering from other inflammatory diseases. In this context, the inflammatory markers IL-1*β* and TNF-*α* and the proportion of necrotic leukocyte/macrophages may be useful as markers of beneficial or harmful levels of physical exercise. 

Stress associated with intense exercise has been reported to induce leukocyte necrosis, but the mechanism of exercise-induced apoptosis is unclear. Some hypotheses may be proposed, such as the body's response to excessive oxidative stress and the increase of cytokines such as tumor necrosis factor alpha, which are important signaling molecules in apoptotic and necrotic pathways [13, 30].

In conclusion, this paper reports that immune activation and inflammatory status over a 24-hour period after a session of intense exercise differs in diabetic rats compared to nondiabetic controls. However, it is important to note that the underlying mechanisms of exercise-induced changes in immune activation and inflammatory responses in diabetic animals are still unknown and lie outside the scope of the present study.

## Figures and Tables

**Table 1:** Blood concentrations of CRP (mg/mL), IgA (*μ*g/mL), IgM (*μ*g/mL), C3 (*μ*g/mL), CINC-2*α*/*β* (pg/mL), MIP-3*α* (pg/mL), L-selectin (pg/mL), IL-1ra (pg/mL), IL-10 (pg/mL), and VEGF-*α* (pg/mL) in non-exercised controls, non-exercised diabetics, exercised control, and exercised diabetic rats measured before, immediately after, and 2 and 24 hours after exercise. Values are presented as mean ± SD of 7 animals per group.

	Control				Diabetic			
	0	IA	2 h	24 h	0	IA	2 h	24 h
CRP (mg/mL)	1.7 ± 0.2	1.7 ± 0.2	1.9 ± 0.1	1.8 ± 0.2	1.7 ± 0.2	1.8 ± 0.3	1.8 ± 0.1	1.7 ± 0.3
C3 (*μ*g/mL)	35 ± 0.9	33 ± 1.6	33 ± 1.2	36 ± 1.1	32 ± 0.7	31 ± 0.8	33 ± 1.3	33 ± 0.8
IgM (*μ*g/mL)	37 ± 2.3	37 ± 3.1	35 ± 1.8	36 ± 2.7	41 ± 0.8	37 ± 6.6	36 ± 1.8	39 ± 4.3
IgA (*μ*g/mL)	27 ± 1.1	27 ± 0.8	26 ± 1.2	27 ± 0.7	28 ± 0.7	26 ± 0.6	26 ± 0.2	28 ± 2.1
CINC-2*αβ* (pg/mL)	17.7 ± 3.0	19.1 ± 2.0	20.5 ± 3.8	18.3 ± 3.2	18.3 ± 4.7	19.1 ± 1.8	18.8 ± 3.4	16.1 ± 3.2
MIP-3*α* (pg/mL)	62.2 ± 11.6	68.8 ± 12.6	70.1 ± 2.7	68.1 ± 18.1	65.9 ± 21.8	60.0 ± 20.8	60.2 ± 19.7	67.6 ± 17.9

## Abbreviations

IgA: Immunoglobulin A
IgM: Immunoglobulin M
CK: Creatine kinase
TNF-*α*: Tumor necrosis factor-alpha
IL-1*β*: Interleukin-1 beta
IL-6: Interleukin-6
ACSM: American College of Sports Medicine
ADA: American Diabetes Association
EASD: European Association for the Study of Diabetes
AHA: American Heart Association
LDH: Lactate dehydrogenase
CINC-3*αβ*: Cytokine-induced neutrophil chemoattractant 3
VEGF: Vascular endothelial growth factor
CRP: C-reactive protein
MIP-3*α*/*β*: Macrophage inflammatory protein-3 alpha/beta
IL-10: Interleukin-10
IL-1ra: Interleukin receptor antagonist 1.
